# FGF9 attenuates osteoarthritis progression through the NRF2/GPX3 antioxidant axis

**DOI:** 10.1302/2046-3758.156.BJR-2025-0365.R1

**Published:** 2026-06-03

**Authors:** Yuan-Shun Lo, Tsung-Ming Chen, Teng-Le Huang, Yu-Chia Liu, Chu-Han Chang, Chia-Yang Chen, Hung-Lun Hsieh, Chun-Hao Tsai, Yi-Xue Yeow, Chia-Jou Wang, Ya-Huey Chen

**Affiliations:** 1 Department of Orthopedic Surgery, China Medical University Beigang Hospital, China Medical University, Yunlin, Taiwan; 2 Department of Orthopedic Surgery, China Medical University Hospital, China Medical University, Taichung, Taiwan; 3 School of Medicine, College of Medicine, China Medical University, Taichung, Taiwan; 4 Department and Graduate Institute of Aquaculture, National Kaohsiung University of Science and Technology, Kaohsiung, Taiwan; 5 College of Biomedical Engineering, China Medical University, Taichung, Taiwan; 6 Department of Biomedical Imaging and Radiological Science, College of Medicine, China Medical University, Taichung, Taiwan; 7 Center for Molecular Medicine, China Medical University Hospital, Taichung, Taiwan; 8 Graduate Institute of Biomedical Sciences, College of Medicine, China Medical University, Taichung, Taiwan; 9 School of Chinese Medicine, College of Chinese Medicine, China Medical University, Taichung, Taiwan; 10 Department of Sports Medicine, China Medical University, Taichung, Taiwan; 11 Program for Cancer Biology and Drug Discovery, College of Medicine, China Medical University, Taichung, Taiwan

**Keywords:** Osteoarthritis, FGF9, Exosome, antioxidant, Osteoarthritis (OA), chondrocytes, cartilage tissues, staining, RNAs, Fibroblast growth factor, pathogenesis, mesenchymal stem cells (MSCs), chondrogenesis

## Abstract

**Aims:**

Osteoarthritis (OA), a prevalent age-related joint disease affecting over 250 million people globally, currently lacks effective disease-modifying treatments. Fibroblast growth factor 9 (FGF9) has shown cartilage-protective effects in post-traumatic OA models, but its role in chondrocyte degeneration and OA pathogenesis remains unclear. This study investigates FGF9’s function in human and murine chondrocytes and its therapeutic potential for OA.

**Methods:**

Gene expression profiling was performed on primary chondrocytes from OA patients and normal controls. Senescence and reactive oxygen species (ROS) levels were assessed by β-galactosidase staining and flow cytometry. FGF9 function was evaluated through short hairpin RNA (shRNA)-mediated knockdown and treatment with FGF9-conditioned media (CM). The impact of FGF9-enriched exosomes on chondrocyte senescence was also examined in vitro. In vivo effects were tested using adenovirus-delivered FGF9 in a destabilization of the medial meniscus (DMM)-induced OA mouse model.

**Results:**

FGF9 expression was significantly downregulated in OA chondrocytes (n = 195) compared to normal (n = 71). FGF9 knockdown elevated ROS levels and senescence via suppression of the NRF2/GPX3 antioxidant axis, while FGF9 promoted chondrogenesis in mesenchymal stem cells. Intra-articular FGF9 gene therapy reduced OA progression in DMM mice. Additionally, FGF9-enriched exosomes reduced senescence in primary chondrocytes in vitro.

**Conclusion:**

FGF9 alleviates OA progression by activating the NRF2/GPX3 pathway, reducing ROS and chondrocyte senescence. These findings support the therapeutic potential of FGF9 and FGF9-enriched exosomes in OA treatment.

Cite this article: *Bone Joint Res* 2026;15(6):632–646.

## Article focus

- To determine the role of fibroblast growth factor 9 (FGF9) in chondrocyte senescence and oxidative stress in osteoarthritis (OA).
- To explore the therapeutic potential of FGF9 and FGF9-loaded exosomes in OA models.

## Key messages

- FGF9 is downregulated in OA and its loss promotes chondrocyte senescence via oxidative stress.
- The FGF9/NRF2/GPX3 axis is a key regulatory pathway in OA pathogenesis.
- FGF9 gene therapy and FGF9-exosomes show therapeutic promise for OA treatment.

## Strengths and limitations

- Identifies a novel antioxidant pathway (FGF9/NRF2/GPX3) critical to chondrocyte ageing in OA.
- Demonstrates in vivo therapeutic efficacy of adenoviral FGF9 and in vitro efficacy of FGF9-enriched exosomes.
- Further in vivo studies are needed to validate the efficacy and safety of FGF9-exosomes for clinical translation.

## Introduction

Osteoarthritis (OA), the most common form of degenerative joint disease, affects approximately 250 million people worldwide.^1^ It is characterized by chronic pain, reduced mobility, and physical limitations that substantially impair quality of life and shorten life expectancy. Current treatments primarily focus on symptom relief, including rest and pain management.^2^ However, effective therapies to prevent or slow OA progression remain limited.

Over time, OA leads to progressive cartilage loss and joint dysfunction, with ageing being a major risk factor due to age-related tissue deterioration.^3^ The disease is characterized by irreversible degeneration of articular cartilage, primarily composed of extracellular matrix produced by chondrocytes. Histological analyses reveal chondrocyte clustering and cartilage volume loss, highlighting the key role of chondrocyte senescence in cartilage integrity and OA progression.^3-7^ Furthermore, age-related imbalance between reactive oxygen species (ROS) production and the antioxidant capacity of chondrocytes contributes to cartilage degradation and chondrocyte death.^8,9^

Fibroblast growth factor 9 (FGF9), a member of the fibroblast growth factor family, plays key roles in development and disease.^10-17^ Mouse models have shown that FGF9 deficiency impairs skeletal growth, while overexpression disrupts chondrocyte proliferation and differentiation.^12,13^ FGF9 activates signalling pathways such as PI3K/Akt, ERK, and SOX9, promoting chondrocyte survival and reducing apoptosis.^18-21^ Under hypoxic conditions, the interplay between FGF9 and ROS regulates signalling pathways which are critical for chondrocyte survival and function.^11,22,23^ These findings suggest that FGF9 plays a role in OA pathogenesis, although the specific antioxidant enzymes and underlying mechanisms remain unclear.

In this study, unbiased analysis revealed significant downregulation of FGF9 in chondrocytes from OA patients. To assess its role in OA pathogenesis and therapeutic potential, we examined FGF9 expression in primary chondrocytes isolated from 195 OA patients over the past decade. We also explored how FGF9 regulates antioxidant genes involved in chondrocyte senescence, and evaluated the therapeutic potential of FGF9, both alone and via exosomes enriched through ectopic FGF9 expression, in the treatment of OA.

## Methods

Human cartilage samples were collected during total knee arthroplasty (TKA) surgeries (Supplementary Material). Chondrocytes were isolated by sequential digestion with pronase and collagenase II in Dulbecco’s Modified Eagle Medium (DMEM)/F12 containing 1% penicillin/streptomycin (Gibco; Thermo Fisher Scientific, USA).^24^ After filtration through a 70 μm sterile mesh, cells were seeded at 1.5 × 10⁵ cells/cm² and cultured in DMEM/F12 supplemented with 10% fetal bovine serum (FBS), 1% penicillin/streptomycin, and 50 μg/ml 2-phospho-L-ascorbic acid. This study was approved by the Research Ethics Committee of China Medical University & Hospital, Taichung, Taiwan.

### Cell culture

Cell culture procedures followed previously published protocols. Human primary chondrocytes were maintained in DMEM/F12 (Gibco). The human chondrocyte cell line C28/I2 (SCC043; Merck, USA) was cultured in high-glucose DMEM (Gibco). ATDC5 mouse chondrogenic precursor cells (RCB0565; RIKEN, Japan) and HEK293 cells were maintained in DMEM/F12, while human mesenchymal stem cells (MSCs) (3A6) were cultured in low-glucose DMEM.

### Lentiviral infection

Lentiviral short hairpin RNA (shRNA) clones were obtained from the National RNAi Core Facility, Academia Sinica (Taiwan). Briefly, cells were seeded at approximately 60% confluency on the first day. On the following day, cells were infected with control or FGF9-specific shRNA lentiviruses including shFGF9-B2 and shFGF9-D1 at a multiplicity of infection (MOI) of 10 in the presence of polybrene (8 μg/ml) for three days.

### Mice OA model

All animal procedures were approved by the Institutional Animal Care and Use Committee (IACUC), China Medical University in accordance with institutional welfare guidelines. We have adhered to the ARRIVE guidelines and have supplied the ARRIVE checklist in the Supplementary Material. OA was induced in the left knee of eight- to ten-week-old male C57BL/6J mice following a standard protocol.^25^ Male mice were used because DMM-induced osteoarthritis is more severe and reproducible in males, while oestrogen-associated effects and oestrous cycle fluctuations in females may increase experimental variability. Therefore, male mice were selected to ensure consistent OA progression and histopathological assessment. Mice were anaesthetized with 1% to 4% isoflurane, and the medial meniscotibial ligament was transected via a medial joint capsule incision to destabilize the meniscus. Sham controls received the same incision without ligament dissection. All mice resumed free movement post-surgery.

### Adenoviral production and injection

*FGF9* complementary DNA (cDNA) was subcloned into the pAdEasy-1 plasmid (adFGF9) using the AdEasy XL system (Agilent Technologies, USA), and transfected into ad293 cells in six-well plates. The virus was amplified through serial passages, ending in a 15 cm dish. A null adenovirus (adnull) generated from pAdEasy-1 served as the control. Adenovirus (1 × 10⁷ or 3 × 10⁷ plaque-forming units (PFUs)) was injected intra-articularly twice weekly, beginning on day 7 post-surgery.^5,26^

### Histological staining

For Alcian Blue staining, 3D pellets of 3A6 cells were fixed in 4% paraformaldehyde, paraffin-embedded, sectioned at 6 μm, and stained with 1% Alcian Blue 8 GX (A5268; Sigma-Aldrich, USA).^10^ For Safranin-O/Fast Green staining, mouse knee joints were fixed in 4% paraformaldehyde, decalcified in 0.5 M EDTA, paraffin-embedded, and sectioned at 90 μm intervals. Sections were deparaffinized, rehydrated, and stained with Safranin-O/Fast Green^10^ or subjected to immunohistochemistry (IHC) using antibodies against FGF9 (AF-273-NA; R&D Systems, USA) and collagen II (ab34712; Abcam, UK). Cartilage thickness and destruction was evaluated using the Osteoarthritis Research Society International (OARSI) scoring system in a blinded manner.^27^

### Bioinformatic analysis

Differential gene expression from microarray and RNA-seq datasets was analyzed using the Database for Annotation, Visualization and Integrated Discovery (DAVID) (v6.7; National Institute of Allergy and Infectious Diseases (NIAID), National Institutes of Health (NIH), USA), with pathways or processes considered significant at fold change ≥ 2 and p ≤ 0.05.^28^

### Statistical analysis

All experimental data were analyzed using GraphPad Prism 9.0 (GraphPad Software, USA) and are presented as mean (SD). Statistical significance was determined by independent-samples *t*-test or one-way analysis of variance (ANOVA), with p < 0.05 considered statistically significant.

## Results

### Recruitment of OA patients

**Fig. 1:**
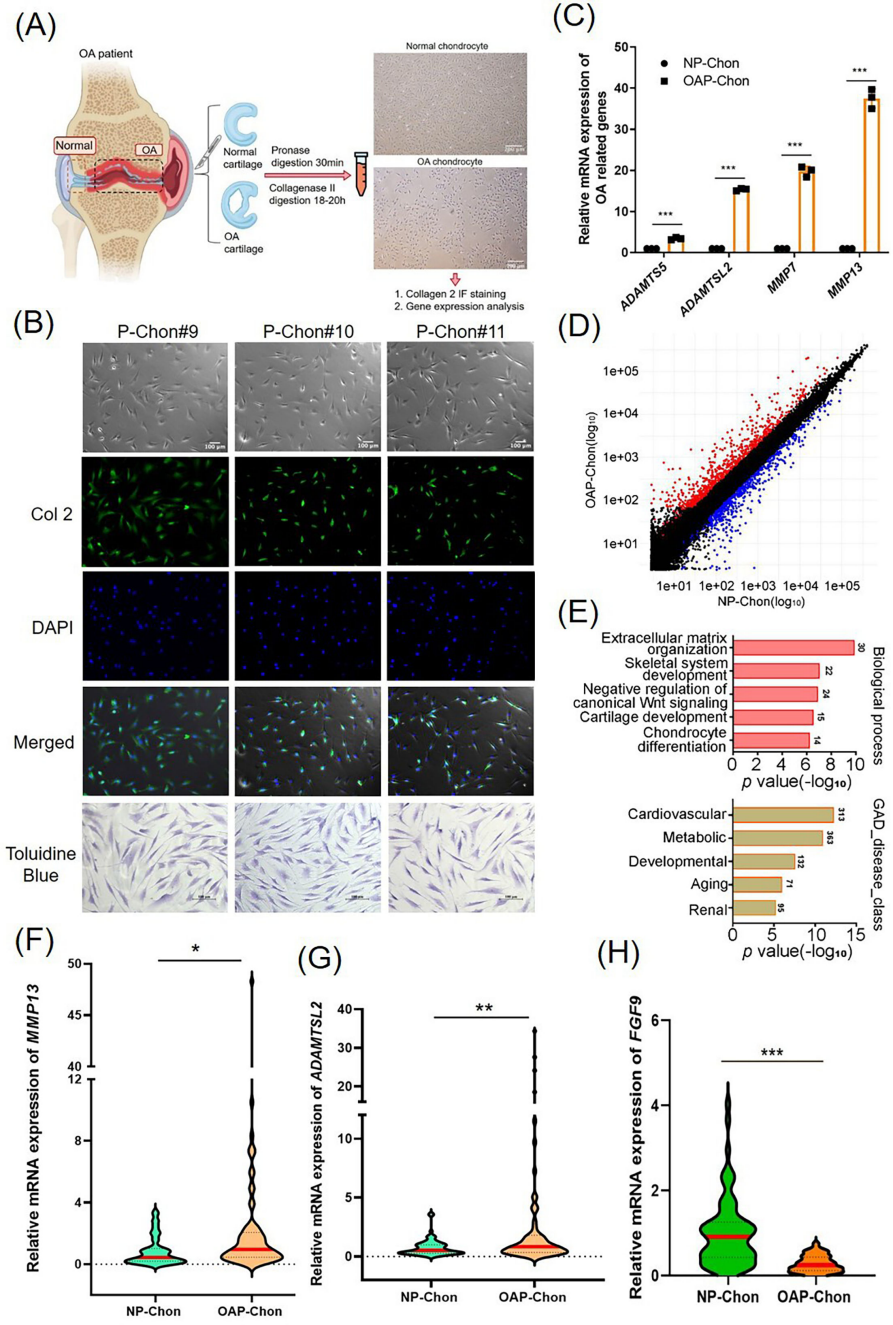
Molecular characterization of osteoarthritis (OA) and normal human primary chondrocytes. a) Schematic illustration of the isolation process for normal (NP-Chon) and OA (OAP-Chon) primary chondrocytes from patient cartilage samples. b) Characterization of isolated chondrocytes using immunofluorescence (IF) staining for collagen II and toluidine blue staining. c) Quantitative reverse transcription polymerase chain reaction (qRT-PCR) analysis of OA-related markers (a disintegrin and metalloproteinase with thrombospondin motifs 5 (*ADAMTS5*)*, ADAMTSL2,* matrix metalloproteinase 7 *(MMP7)*, and *MMP13*) in NP-Chon and OAP-Chon cells. d) Scatter plot showing the global gene expression profiles of NP-Chon and OAP-Chon cells; 698 genes were upregulated (red) and 560 downregulated (blue) in OAP-Chon with a ≥ twofold change. e) Gene Ontology (GO) term enrichment analysis of differentially expressed genes in OAP-Chon, focusing on biological processes (upper panel) and disease associations (lower panel). f) and g) qRT-PCR validation of *MMP13* and *ADAMTSL2* messenger RNA (mRNA) expression in NP-Chon (n = 68) and OAP-Chon (n = 70). h) qRT-PCR analysis of fibroblast growth factor 9 (FGF9) mRNA expression in NP-Chon (n = 71) and OAP-Chon (n = 195). Error bars represent mean (SD). *p < 0.05, **p < 0.01, ***p < 0.001, independent-samples *t*-test. GAD, Genetic Association Database.

Human primary chondrocytes were isolated from normal and OA cartilage tissues (Figure 1a). Patients with unilateral severe knee OA (Kellgren–Lawrence grade 3 to 4)^29^ undergoing TKA and showing no patellofemoral joint involvement (Kellgren–Lawrence grade 0) were recruited. Prior to TKA, intact patellofemoral cartilage (Outerbridge grade 0)^30^ was arthroscopically biopsied and designated as normal primary chondrocytes (NP-Chon). Following TKA, cartilage from weightbearing regions showing fissures or subchondral bone exposure was collected to isolate OA primary chondrocytes (OAP-Chon). The study included 195 OAP-Chon and 71 NP-Chon samples (mean age 72 years (SD 10.1)) (Supplementary Table i). Chondrocyte identity was confirmed by immunofluorescence staining for collagen II (cytoplasmic green fluorescence) and toluidine blue staining, which showed typical spindle-shaped morphology (Figure 1b).

To validate differential gene expression between OAP-Chon and NP-Chon, we assessed key OA markers (*ADAMTS5*, *ADAMTSL2*, matrix metalloproteinase 7 (*MMP7)*, and *MMP13*) by qRT-PCR. All markers were significantly upregulated in OAP-Chon compared to NP-Chon (p < 0.001, independent-samples *t*-test; Figure 1c), confirming the distinct molecular profile of OA chondrocytes and supporting their pathological relevance.

### FGF9 is downregulated in chondrocytes from OA patients

To characterize gene expression profiles in OAP-Chon and NP-Chon, cDNA microarray analysis was performed. A scatter plot revealed 698 upregulated and 560 downregulated genes in OAP-Chon (≥ twofold change; Figure 1d). Gene Ontology (GO) analysis of the 1,258 differentially expressed genes showed enrichment in extracellular matrix organization and skeletal and cartilage development (Figure 1e, upper panel), as well as disease categories including cardiovascular, metabolic, and ageing-related conditions (Figure 1e, lower panel). Additional GO enrichment confirmed associations with cellular components, molecular functions, and Kyoto Encyclopedia of Genes and Genomes (KEGG) pathways relevant to OA (Supplementary Figure aa).

To assess the clinical relevance of microarray-identified genes, we examined the expression of *MMP13*, *ADAMTSL2*, and *FGF9* (key regulators of cartilage development) using qRT-PCR. *MMP13* (p = 0.020; Figure 1f) and *ADAMTSL2* (p = 0.006; Figure 1g) were significantly upregulated in OAP-Chon (n = 70 to 195) compared to NP-Chon (n = 68 to 71), while FGF9 expression was markedly reduced (p < 0.001, independent-samples *t*-test; Figure 1h). These findings corroborate the microarray data and underscore the clinical significance of *MMP13*, *ADAMTSL2*, and *FGF9* in OA pathogenesis.

### FGF9 is involved in cellular senescence of chondrocytes

**Fig. 2:**
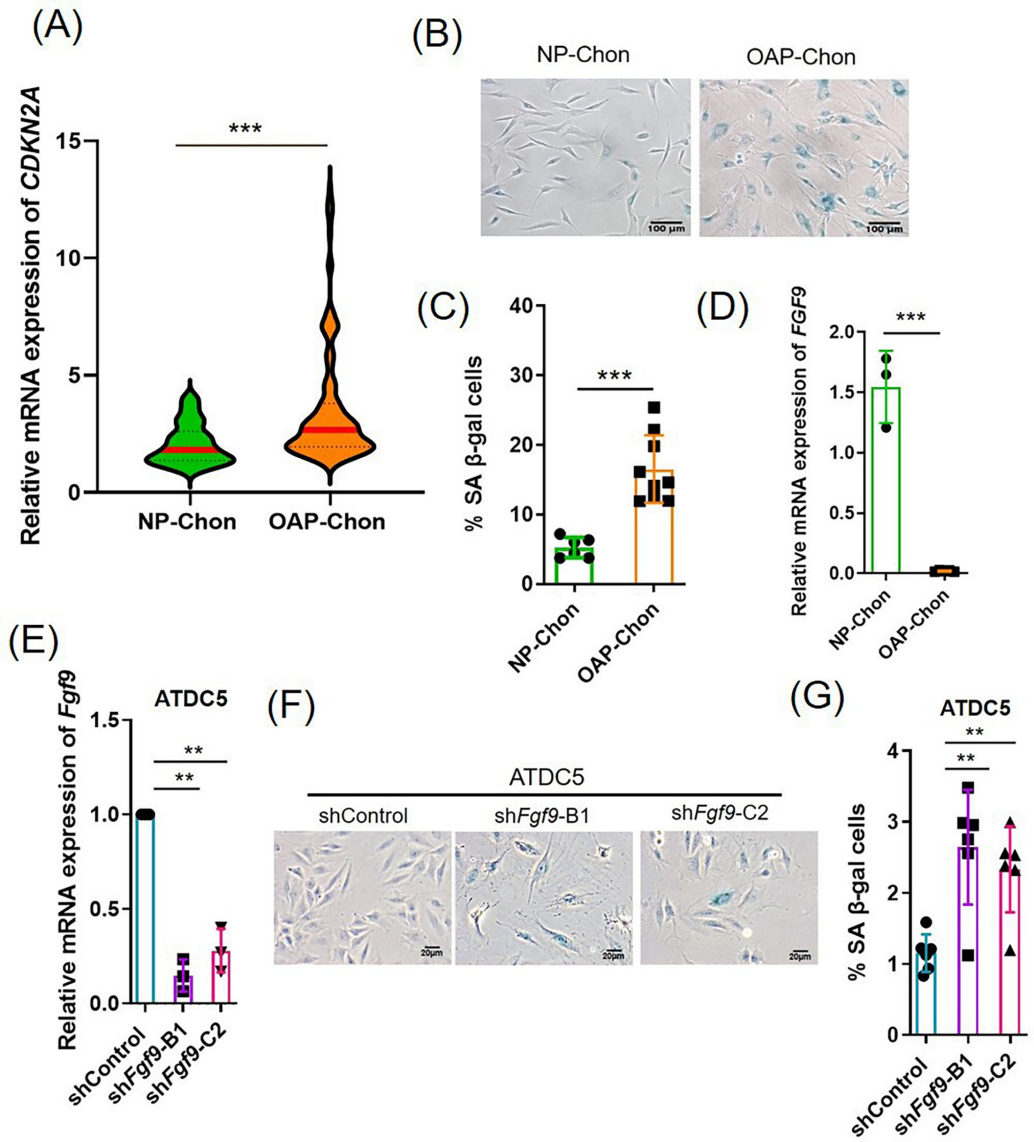
Fibroblast growth factor 9 (FGF9) is downregulated in osteoarthritis (OA) chondrocytes and regulates cellular senescence. a) Quantitative reverse transcription polymerase chain reaction (qRT-PCR) analysis of cyclin-dependent kinase inhibitor 2A (*CDKN2A*) messenger RNA (mRNA) expression in normal (NP-Chon) (n = 71) and OA primary chondrocyte (OAP-Chon) (n = 195) cells. b) Representative images of senescence-associated β-galactosidase (SA-β-Gal) staining in NP-Chon and OAP-Chon cells; senescent cells appear blue. c) Quantification of SA-β-Gal–positive senescent cells. d) qRT-PCR analysis of *FGF9* mRNA expression in NP-Chon and OAP-Chon cells. e) qRT-PCR analysis of *Fgf9* expression in ATDC5 cells following lentiviral short hairpin RNA (shRNA)-mediated knockdown. f) Representative SA-β-Gal staining images of *Fgf9*-depleted ATDC5 cells. g) Quantification of senescent ATDC5 cells following *Fgf9* knockdown. Error bars represent mean (SD). **p < 0.01, ***p < 0.001, independent-samples *t*-test.

Ageing is a major risk factor for OA and targeting age-related chondrocyte senescence holds therapeutic promise. To confirm senescence in OA chondrocytes, we measured cyclin-dependent kinase inhibitor 2A (*CDKN2A*) (p16) mRNA and β-galactosidase activity. Both were significantly elevated in OAP-Chon compared to NP-Chon (*CDKN2A*: n = 195 vs 71, p < 0.001; β-galactosidase activity: p < 0.001) (Figures 2a to 2c), indicating a senescent phenotype. qRT-PCR further revealed reduced *FGF9* mRNA in OAP-Chon (p < 0.001, independent-samples *t*-test) (Figure 2d). Expression of *FGFR2* and *FGFR3* was comparable between NP-Chon (n = 20) and OAP-Chon (n = 87) (Supplementary Figure ab), suggesting that FGF9 downregulation, not receptor availability, is the key alteration in OA-related FGF9 signalling.

To investigate FGF9’s function in chondrocytes, we knocked down Fgf9 in murine chondrogenic precursor cells (ATDC5) using lentiviral shRNA (Figure 2e). Fgf9 depletion significantly increased cellular senescence (p = 0.001, independent-samples *t*-test; Figures 2f and 2g), revealing a negative correlation between Fgf9 expression and senescence. These results identify FGF9 as a key regulator of chondrocyte senescence and support its involvement in OA pathogenesis.

### FGF9 plays an important role in regulating ROS levels in chondrocytes and influencing OA progression

**Fig. 3:**
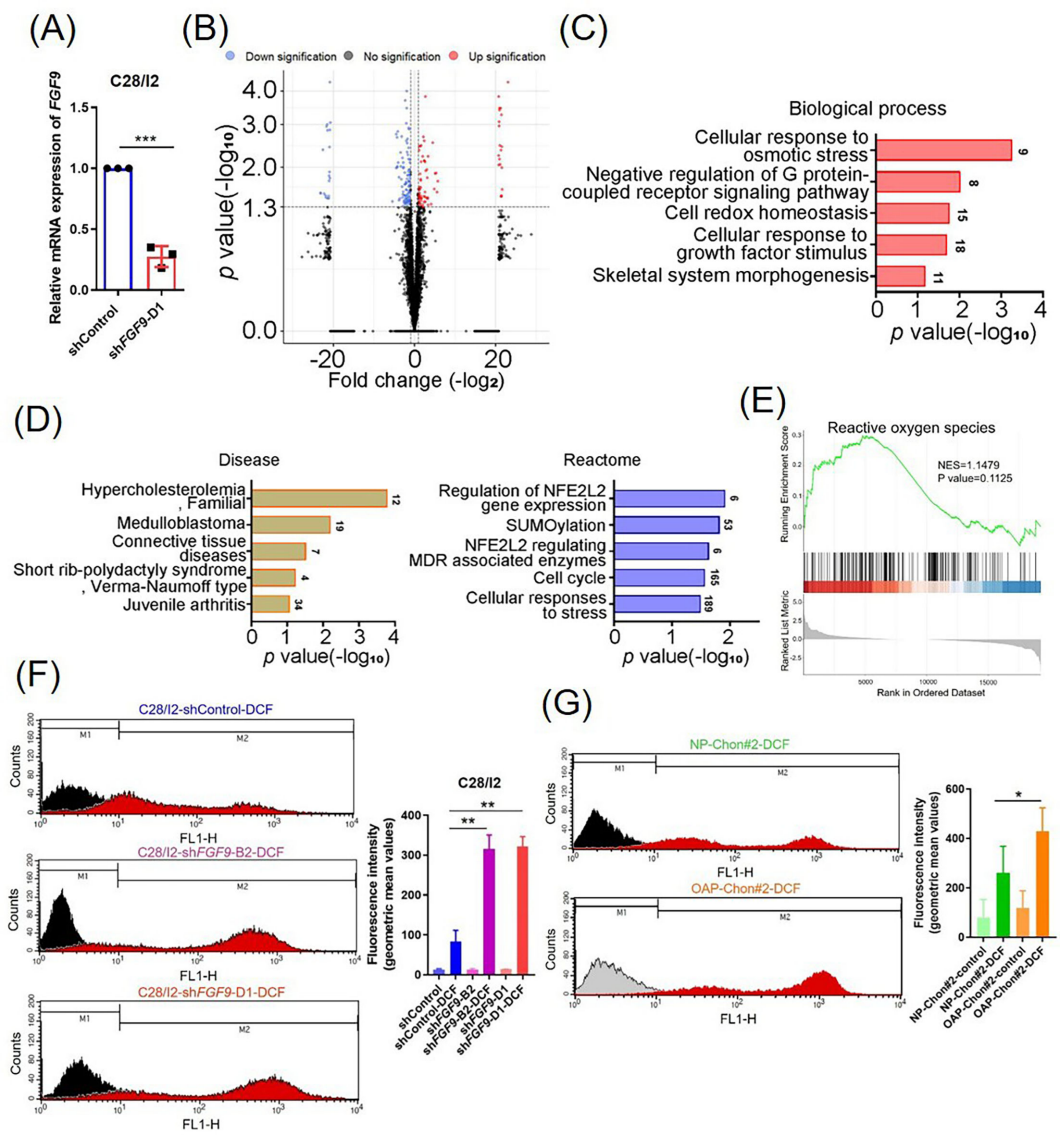
Transcriptomic analysis reveals oxidative stress regulation by fibroblast growth factor 9 (FGF9) in chondrocytes. a) Confirmation of FGF9 knockdown in C28/I2 cells using control and specific FGF9 short hairpin RNA (shRNA) (shRNA-D1). b) Volcano plot of global gene expression changes in FGF9-depleted C28/I2 cells; upregulated and downregulated genes (≥ twofold change) are shown in red and blue, respectively. c) Gene Ontology (GO) analysis of enriched biological processes in FGF9-depleted cells. d) GO enrichment of differentially expressed genes categorized by disease relevance (left) and reactome pathways (right). e) Normalized enrichment scores (NES) for reactive oxygen species (ROS)-related genes in FGF9-depleted cells. f) ROS levels measured by dichlorofluorescein (DCF) fluorescence in C28/I2 cells with control and two distinct FGF9 shRNAs (shFGF9-B2 and shFGF9-D1) (left); quantification of mean fluorescence intensity (right). g) DCF-based ROS detection in normal (NP-Chon) and OA primary chondrocytes (OAP-Chon) (left); quantification of ROS levels (right). Error bars represent mean (SD). *p < 0.05, **p < 0.01, ***p < 0.001, independent-samples *t*-test. MDR, multidrug resistance; mRNA, messenger RNA.

To further explore FGF9’s role in chondrocyte senescence, RNA-seq was performed on FGF9-depleted C28/I2 cells (p < 0.01; Figure 3a), revealing 3,805 DEGs (≥ twofold, p < 0.05, independent-samples *t*-test; Figure 3b). GO analysis indicated enrichment in redox homeostasis and osmotic stress (Figure 3c), while disease associations included skeletal disorders (Figure 3d, left). Reactome analysis highlighted pathways such as NFE2L2 signalling and stress response (Figure 3d, right). Among the DEGs, 364 FGF9-regulated genes were linked to redox balance and ROS. Enrichment analysis showed that FGF9 depletion was positively associated with oxidative phosphorylation and ROS accumulation (Figure 3e, Supplementary Figure ac).

To evaluate FGF9’s role in ROS regulation, flow cytometry analysis revealed that FGF9 depletion by specific shRNAs (B2 and D1) in C28/I2 cells significantly increased DCF fluorescence intensity compared to control cells, indicating elevated ROS levels (p = 0.006, independent-samples *t*-test; Figure 3f). Similarly, OAP-Chon cells exhibited higher ROS levels than NP-Chon (p = 0.019, independent-samples *t*-test; Figure 3g). These results highlight FGF9’s critical role in modulating ROS in both cell models.

### Reduction of FGF9 promotes ROS via attenuation of NRF2/GPX3

**Fig. 4:**
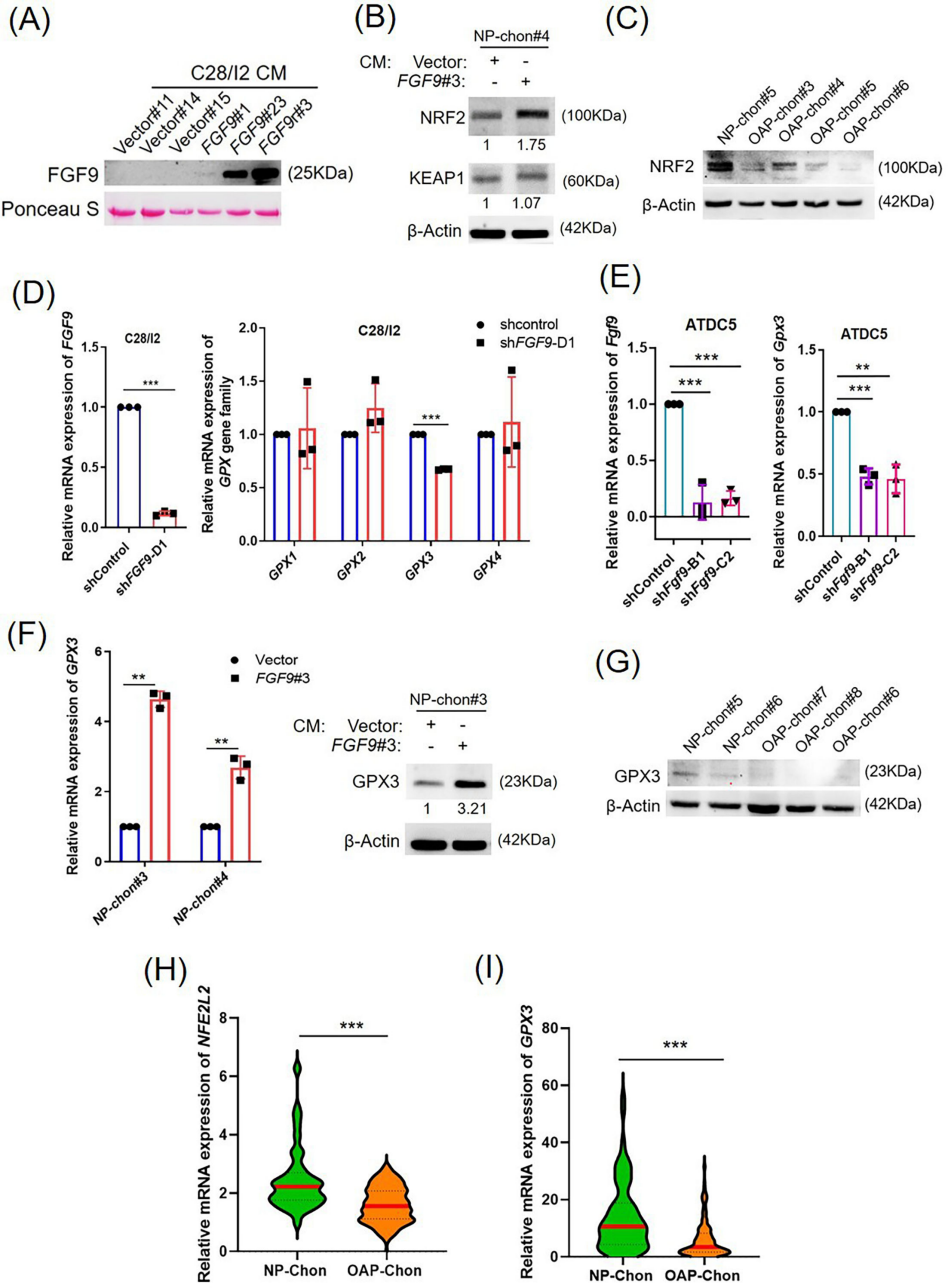
Fibroblast growth factor 9 (FGF9) regulates NRF2/GPX3 expression in chondrocytes. a) Western blot analysis of FGF9 levels in conditioned media (CM) from C28/I2 cells. b) Western blot analysis of NRF2, and KEAP1 in normal primary chondrocytes (NP-Chon) cultured with FGF9-CM; β-actin was used as a loading control. c) Western blot analysis of NRF2 in NP-Chon and osteoarthritis (OA) primary chondrocytes (OAP-Chon). d) Left: Quantitative reverse transcription polymerase chain reaction (qRT-PCR) analysis of FGF9 messenger RNA (mRNA) expression in C28/I2 cells infected with shFGF9-D1. Right: qRT-PCR analysis of GPX1–4 expression in FGF9-depleted C28/I2 cells. e) qRT-PCR analysis of Fgf9 (left) and Gpx3 (right) expression in ATDC5 cells infected with lentiviral control or short hairpin RNA (shRNA). f) Left: qRT-PCR analysis of GPX3 mRNA expression in NP-Chon cells treated with vector control or FGF9-conditioned medium (FGF9-CM). Right: Western blot analysis of GPX3 protein expression in NP-Chon cells treated with vector control or FGF9-CM. g) Western blot analysis of GPX3 protein expression in NP-Chon and OAP-Chon cells. h) and i) qRT-PCR analysis of NFE2L2 and GPX3 expression levels in NP-Chon (n = 71) and OAP-Chon (n = 195) samples, presented as violin plots. Error bars represent mean (SD). **p < 0.01, ***p < 0.001, independent-samples *t*-test.

RNA-seq analysis implicated *NFE2L2* (NRF2) in FGF9-mediated ROS regulation via the KEAP1-NRF2 pathway. Conditioned media (CM) from C28/I2 stable clones expressing vector (#11, #14, #15) or FGF9 (#23, #3) were collected for western blot analysis (Figure 4a). Normal chondrocytes treated with CM from FGF9#3-expressing cells exhibited increased NRF2 expression without changes in KEAP1 compared to those treated with vector#11 CM, as shown by western blot (Figure 4b), suggesting that FGF9 reduces ROS levels by upregulating NRF2. Additionally, NRF2 protein expression was significantly reduced in OAP-Chon samples (Figure 4c), highlighting its role in OA pathogenesis.

Glutathione peroxidases (GPXs), key NRF2 targets, maintain redox homeostasis in OA.^31,32^ To identify NRF2-regulated GPXs, we assessed GPX mRNA expression in FGF9-depleted C28/I2 cells, where GPX3 was significantly reduced (p < 0.001, independent-samples *t*-test; Figure 4d). Similar results were observed in Fgf9-depleted ATDC5 cells (p < 0.001, independent-samples *t*-test; Figure 4e). In contrast, FGF9-CM treatment increased GPX3 mRNA and protein levels (Figure 4f). GPX3 was also markedly downregulated in OAP-Chon samples, consistent with reduced NRF2 expression (Figures 4g to 4i). These findings suggest that FGF9 regulates ROS through the NRF2/GPX3 pathway, contributing to OA pathogenesis.

### FGF9 promotes GPX3 expression through ARE located at GPX3 promoter

**Fig. 5:**
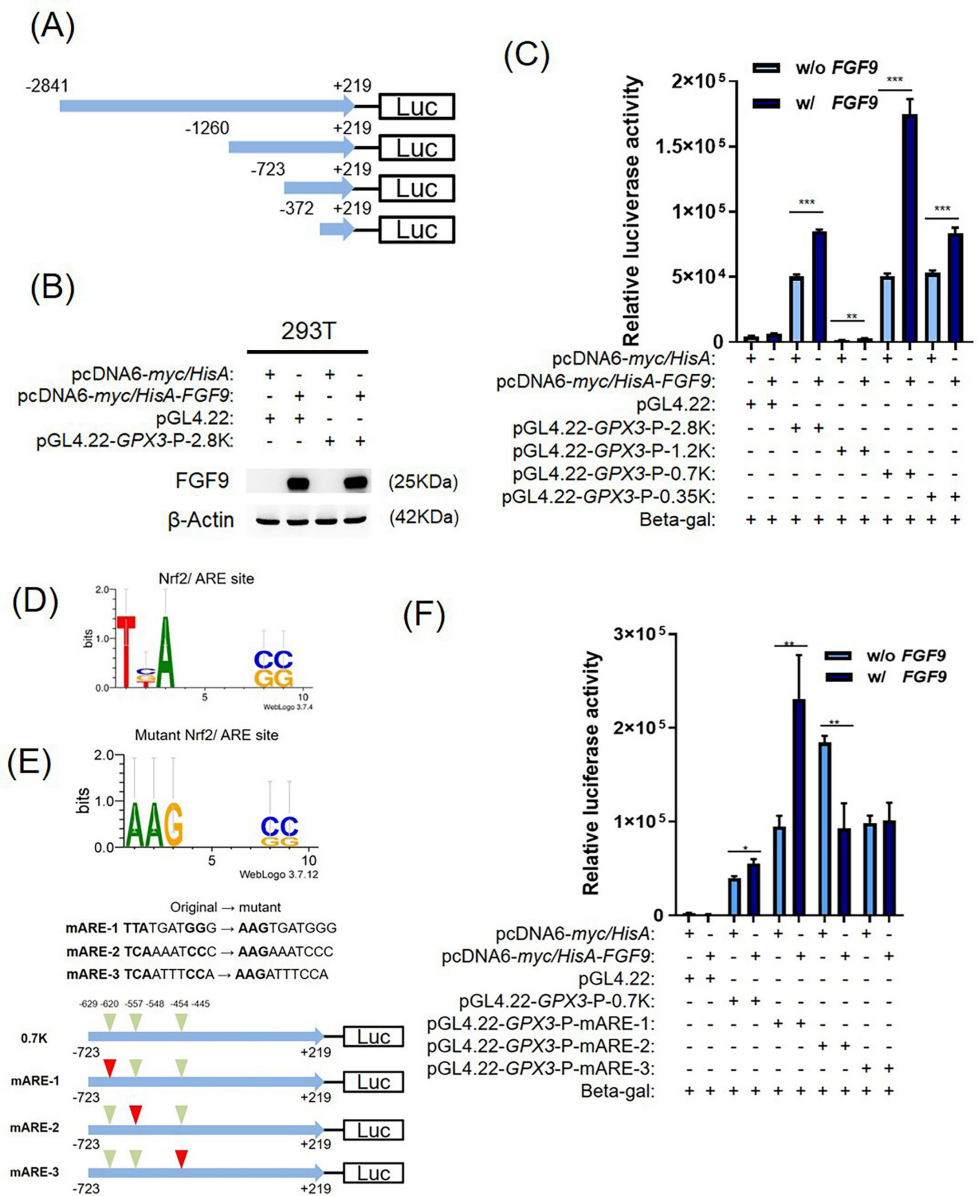
Fibroblast growth factor 9 (FGF9) regulates GPX3 transcription through NRF2-responsive elements in the promoter region. a) Schematic diagram of GPX3 luciferase reporter constructs containing the full-length (-2,841 to +219 bp) and serial deletions of the promoter region. b) Western blot confirming ectopic expression of FGF9 used in the GPX3 promoter assay. c) Luciferase reporter assay of GPX3 promoter constructs in 293 cells with or without ectopic FGF9 expression. d) and e) Schematic of predicted NRF2/ARE binding sites located between -723 and +219 bp of the GPX3 promoter, with sequences of three mutated ARE sites (mARE-1, mARE-2, mARE-3). f) Luciferase reporter assay of wild-type and ARE-mutated GPX3 promoter constructs in 293 cells with or without ectopic FGF9 expression. Error bars represent mean (SD). *p < 0.05, **p < 0.01, ***p < 0.001, independent-samples *t*-test.

NRF2 activates gene expression by binding antioxidant response elements (ARE) in promoters. To assess whether FGF9 regulates NRF2-dependent GPX3 expression, luciferase constructs with *GPX3* promoter deletions (-2,841 to -372 bp; Figure 5a) were tested in 293T cells. FGF9 enhanced *GPX3* promoter activity, with the -723 bp fragment showing the strongest response (p < 0.001, independent-samples *t*-test; Figures 5b and 5c), indicating that the -723 to -372 bp region contains key NRF2-responsive elements.

PROMO analysis identified three ARE sites (ARE-1, -2, -3) within the *GPX3* promoter (Figure 5d). To pinpoint key NRF2 binding sites, site-directed mutagenesis was performed within the -723 to -372 bp region (Figure 5e). Luciferase assays showed that mutations in ARE-2 and ARE-3 were significantly reduced or without affected FGF9-induced promoter activity compared to the wild-type (p = 0.005, independent-samples *t*-test; Figure 5f), indicating that these sites are essential for FGF9-mediated *GPX3* activation.

### FGF9 prevents senescence of chondrocytes and may promote chondrogenesis

**Fig. 6:**
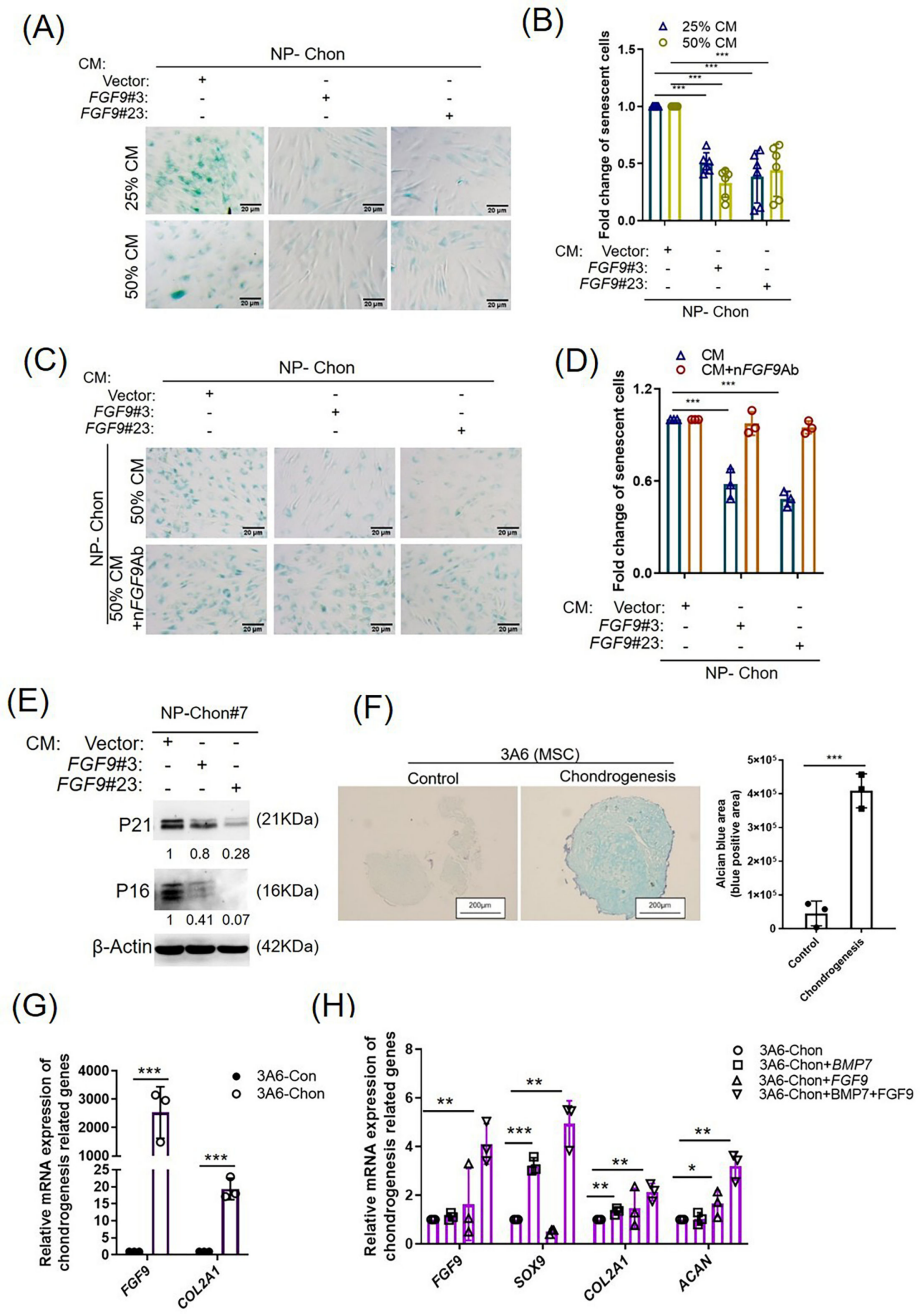
Fibroblast growth factor 9 (FGF9) reduces chondrocyte senescence and promotes chondrogenesis in human 3A6 cells. a) Representative images of senescence-associated β-galactosidase (SA-β-Gal) staining in normal primary chondrocytes (NP-Chon) treated with FGF9-conditioned medium (CM). Senescent cells are indicated by blue staining. b) Quantification of SA-β-Gal–positive cells. Results are normalized to vector control and presented as mean (SD). c) SA-β-Gal staining of NP-Chon cultured in FGF9-CM with or without neutralizing FGF9 antibody (nFGF9Ab). Senescent cells are indicated in blue. d) Quantification of SA-β-Gal–positive cells from Figure 6c, normalized to vector control and shown as mean (SD). e) Western blot analysis of NP-Chon treated with FGF9-CM or control-CM for 48 hours. f) Representative Alcian Blue staining and quantification of 3A6 cell pellets after 21-day chondrogenic induction. g) and h) Quantitative reverse transcription polymerase chain reaction (qRT-PCR) analysis of chondrogenic markers (*FGF9, SOX9,* collagen type II alpha 1 (*COL2A1*)*,* aggrecan (*ACAN*)) in 3A6 cell pellets. Error bars represent mean (SD). *p < 0.05, **p < 0.01, ***p < 0.001, independent-samples *t*-test. mRNA, messenger RNA.

FGF9-CM treatment significantly reduced β-gal-positive senescent cells in normal primary chondrocytes compared to vector-CM (p < 0.001, independent-samples *t*-test; Figures 6a and 6b). Neutralization with FGF9 antibody (nFGF9Ab) reversed this effect, increasing senescence (p < 0.001, independent-samples *t*-test; Figures 6c and 6d). Western blot analysis showed reduced p16 and p21 levels following FGF9-CM treatment (Figure 6e). These findings suggest that FGF9 attenuates chondrocyte senescence and ROS via activation of the NRF2/GPX3 axis.

FGF9 has been shown to promote chondrogenesis,^10^ consistent with RNA-seq data linking it to skeletal morphogenesis (Figure 3c). To investigate this, 3A6 MSCs were induced into chondrogenic lineages (Figure 6f). After three weeks, qRT-PCR revealed elevated *FGF9* and *COL2A1* expression (p < 0.001, independent-samples *t*-test; Figure 6g). Co-treatment with BMP7 (a chondrogenic promoter) and FGF9 further enhanced expression of chondrogenic markers (*FGF9*, *SOX9*, *COL2A1*, *ACAN*; Figure 6h), indicating that combined FGF9 and BMP7 treatment produces a synergistic enhancement of chondrogenic differentiation in MSCs.^33,34^ However, the precise role of FGF9-mediated chondrogenesis in OA has yet to be fully elucidated and will require further investigation.

### FGF9 reduces the degeneration of chondrocytes in mice OA model

**Fig. 7:**
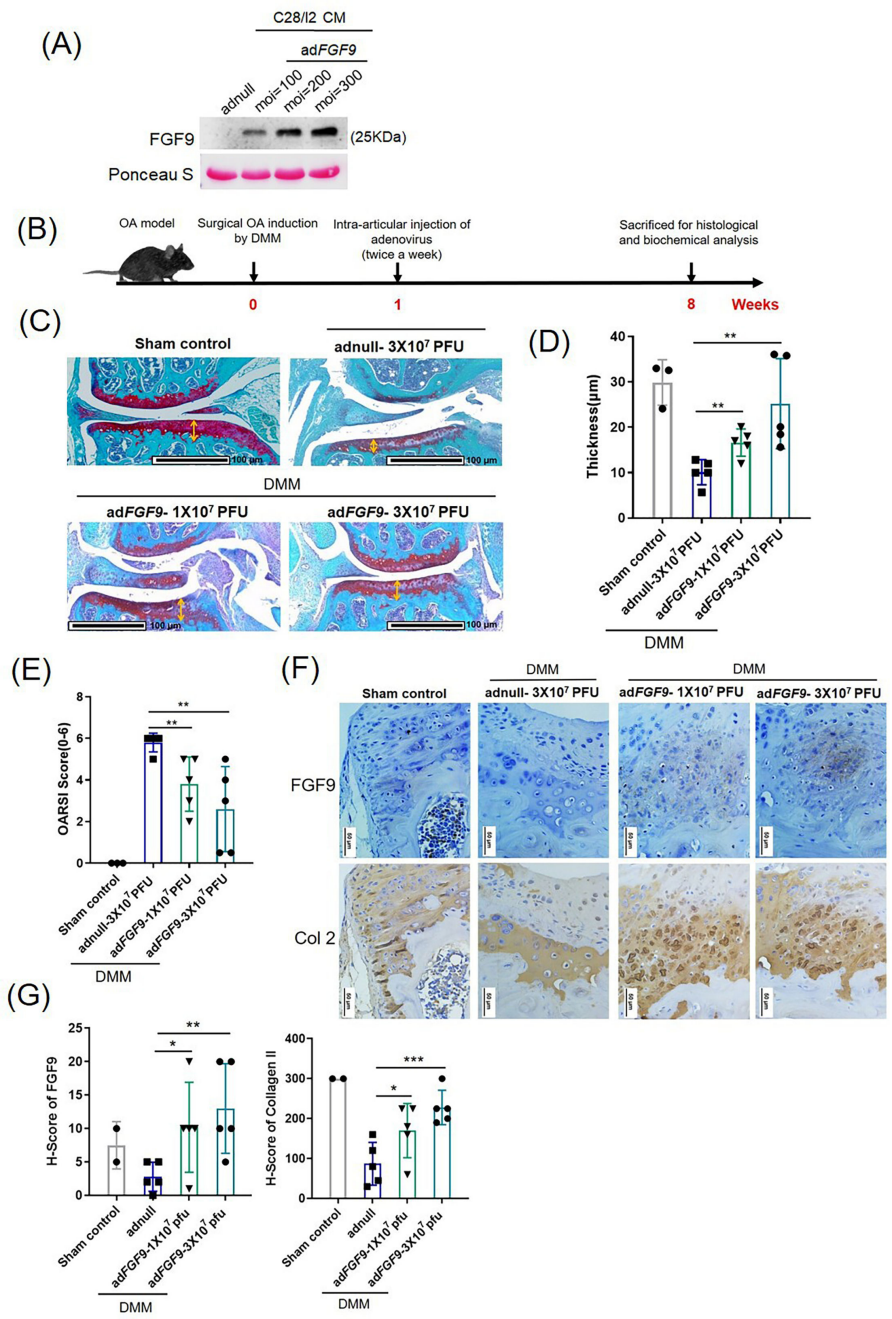
Intra-articular delivery of adenoviral fibroblast growth factor 9 (FGF9) attenuates cartilage degeneration in a destabilization of the medial meniscus (DMM)-induced osteoarthritis (OA) model. a) Western blot analysis of conditioned media (CM) from C28/I2 cells infected with adFGF9 or adnull adenovirus. b) Schematic illustration of the adenoviral treatment protocol in DMM mice. c) Safranin-O/Fast Green staining of knee cartilage from sham-operated and DMM mice treated with adenovirus. Red-stained areas indicate preserved cartilage. d) Double-headed arrows indicate the thickness of the articular cartilage. Quantitative analysis of cartilage thickness is presented in the accompanying bar graph. Error bars represent mean (SD). **p < 0.01. e) Quantification of cartilage degeneration using the Osteoarthritis Research Society International (OARSI) scoring system. The bars represent the OARSI scores. Error bars represent mean (SD). **p < 0.01. f) Immunohistochemistry (IHC) analysis of FGF9 and collagen II (Col 2) expression in knee cartilage from sham-operated and DMM mice treated with adenovirus. g) Statistical analysis of H-score of collagen II and FGF9. Error bars represent mean (SD). *p < 0.05, **p < 0.01, ***p < 0.001, independent-samples *t*-test.

To assess the therapeutic role of FGF9 in OA, we administered intra-articular injections of adenovirus-delivered FGF9 in a destabilization of the medial meniscus (DMM) mouse model. Dose-dependent overexpression of adenovirus-delivered FGF9 was confirmed in C28/I2 cells (Figure 7a). As outlined in Figure 7b, male C57BL/6J mice (8 to 10 weeks old) underwent DMM surgery and were divided into three groups: adnull (3 × 10⁷ PFU), adFGF9 (1 × 10⁷ PFU), and adFGF9 (3 × 10⁷ PFU). Starting one week post-surgery, adenoviral injections were given twice weekly for eight weeks.^5,26^

Safranin-O/Fast Green staining was used to evaluate cartilage preservation and degeneration, quantified using the thickness of cartilage and OARSI scoring system (maximum score: 6.0).^27^ AdFGF9-treated mice exhibited significantly lower OARSI scores and increased cartilage thickness in a dose-dependent manner compared to controls (p < 0.01, independent-samples *t*-test; Figures 7c to 7e). The expression of FGF9 and collagen II (a cartilage-specific marker) in DMM-induced mice treated with Adnull or AdFGF9 adenovirus was examined by IHC (Figures 7f and 7g). Ectopic FGF9 expression markedly attenuated OA progression in DMM-induced mice.

### FGF9 exosome treatment significantly reduced senescence in primary chondrocytes

**Fig. 8:**
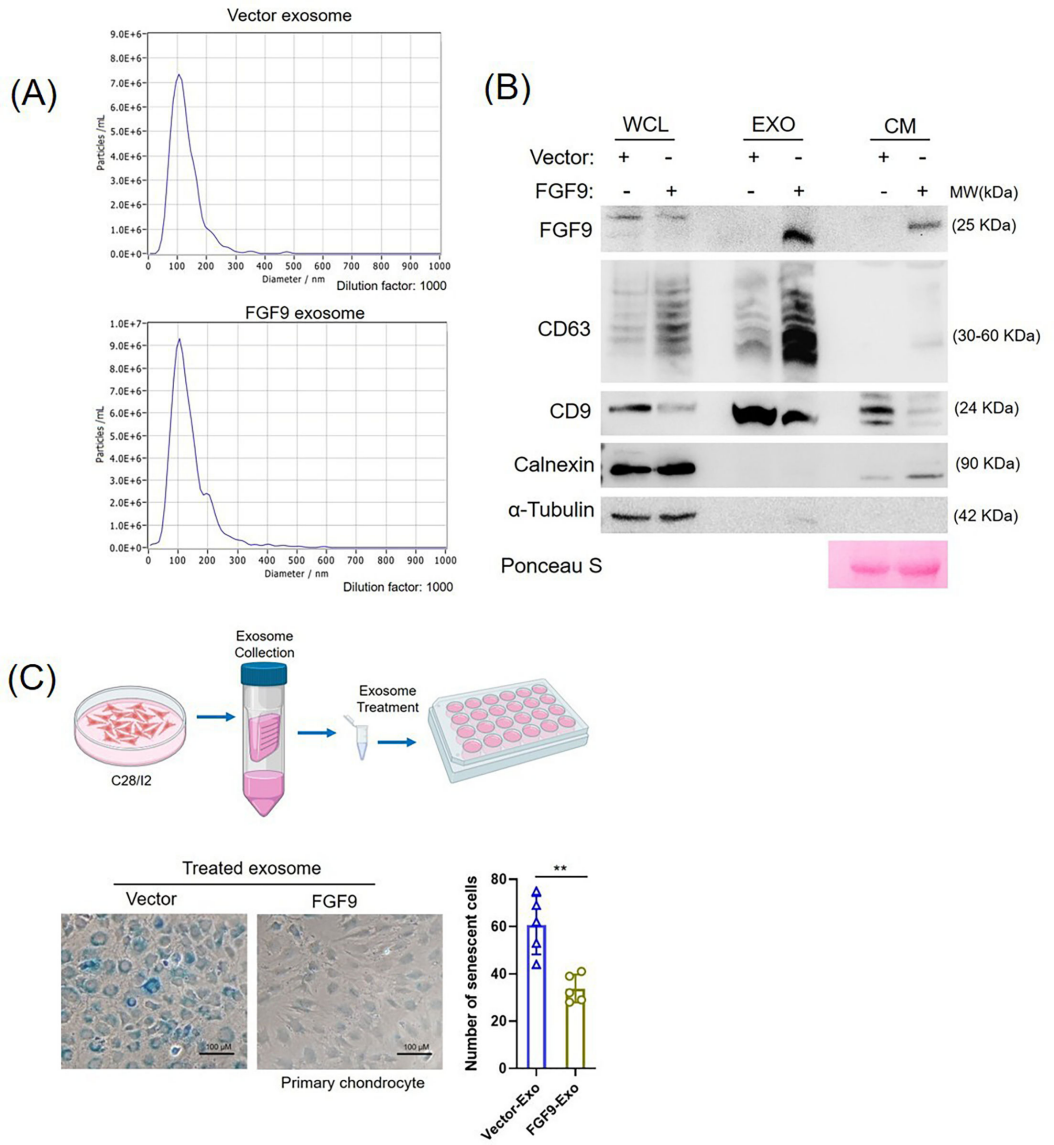
Fibroblast growth factor 9 (FGF9)-exosome attenuates the senescence of primary chondrocytes. a) Nanoparticle tracking analysis (NTA) of exosomes isolated from C28/I2 chondrocytes stably expressing vector control or FGF9. b) Western blot analysis of FGF9, exosomal markers (CD63, CD9), and negative marker (calnexin) in whole cell lysates (WCL), exosomes (EXO), and conditioned media (CM). α-Tubulin was used as a loading control. c) Senescence-associated β-galactosidase (SA-β-Gal) staining of primary chondrocytes treated with vector or FGF9-exosomes (7 × 10⁹ particles) for one week. Representative images of senescent cells (blue staining, left panel) and quantification of SA-β-Gal–positive cells (right panel) are shown. Error bars represent mean (SD). **p < 0.01, independent-samples *t*-test.

**Fig. 9:**
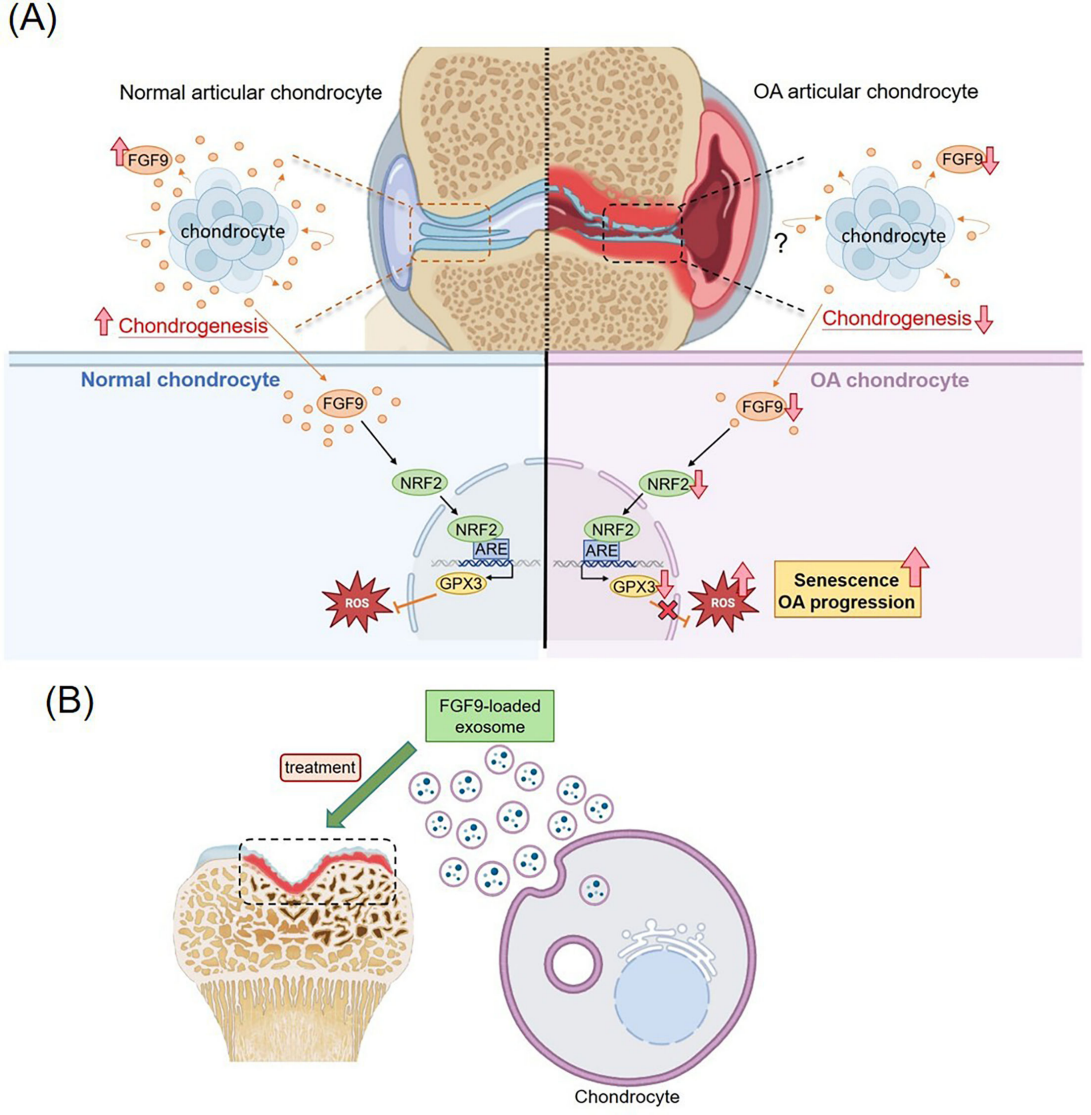
Schematic summary of the proposed fibroblast growth factor 9 (FGF9)-mediated mechanism in osteoarthritis (OA) and its therapeutic potential**.** a) In normal chondrocytes, FGF9 activates the NRF2/GPX3 axis, reducing reactive oxygen species (ROS) levels and preventing senescence. In OA, reduced FGF9 expression leads to ROS accumulation, increased chondrocyte senescence, and cartilage degeneration. b) Exosomes derived from FGF9-overexpressing chondrocytes may deliver therapeutic FGF9, attenuating senescence and offering a potential treatment strategy for OA.

Given the therapeutic potential of exosomes in OA,^35^ we investigated whether exosomes from FGF9-overexpressing chondrocytes are enriched in FGF9. Exosomes were isolated from C28/I2 cells stably expressing vector or FGF9 and characterized by nanoparticle tracking analysis (NTA; Figure 8a) and western blotting using exosomal markers CD63 and CD9, with calnexin as a negative control (Figure 8b). FGF9 was enriched in exosomes compared to whole cell lysates (WCL) and CM (Figure 8b). To assess their therapeutic effect, FGF9-enriched and control exosomes were applied to primary chondrocytes. FGF9 exosomes significantly reduced cellular senescence compared to controls (Figure 8c), suggesting that FGF9-loaded chondrocyte-derived exosomes may serve as a promising OA therapy (Figure 9).

## Discussion

In this study, we identified distinct gene expression profiles between normal and OA human chondrocytes, highlighting FGF9 as a potential therapeutic target. FGF9 was downregulated in OA, and its loss promoted chondrocyte senescence and ROS accumulation by suppressing the NRF2/GPX3 axis. Restoration of FGF9, via adenoviral delivery or FGF9-enriched exosomes, attenuated OA progression in a DMM mouse model and reduced chondrocyte senescence (Figures 9a and 9b). These findings suggest that FGF9 mitigates OA through antioxidant regulation and holds promise as a therapeutic strategy.

We are the first to identify GPX3 as a novel downstream target of the FGF9/NRF2 pathway, mediating FGF9’s antioxidant effects in chondrocytes. As the only extracellular GPX family member, GPX3 reduces lipid hydroperoxides and supports cartilage homeostasis.^36^ Reduced GPX3 expression, as seen in Kashin-Beck disease, has been linked to increased chondrocyte apoptosis.^36^ Our findings show that sustained FGF9 expression activates the NRF2/GPX3 axis, and these results uncover FGF9’s therapeutic potential in regulating cartilage regeneration and OA progression.

Zhou et al^18^ reported reduced FGF9 expression in OA cartilage, consistent with our findings. In post-traumatic OA (C3H/HeJ mice), recombinant FGF9 reduced cartilage degeneration and hypertrophic markers (collagen X, MMP13), but increased osteophyte formation, possibly due to MSC-driven chondrogenesis.^18^ These effects appear strain- and model-dependent. In contrast, our eight-week study using the widely adopted DMM-induced OA model in C57BL/6 mice employed adenovirus-mediated FGF9 delivery, enabling sustained local expression and potentially supporting more stable cartilage repair.

Exosomes have emerged as key mediators of intercellular communication, carrying proteins, lipids, and nucleic acids, including noncoding RNAs, which hold promise as biomarkers and therapeutic agents in OA.^37^ In this study, we found that FGF9 can be enriched in exosomes derived from FGF9-overexpressing chondrocytes (C28/I2). However, the therapeutic efficacy of FGF9-exosomes may vary depending on their cellular origin, such as chondrocytes or MSCs, and should be further evaluated using both in vitro and in vivo models.^38-40^ Moreover, the therapeutic potential of FGF9-exosomes as treatment agents or delivery vehicles for OA warrants further investigation.

In conclusion, we define the FGF9/NRF2/GPX3 axis as a key regulator in OA chondrocyte pathogenesis and propose adenoviral FGF9 and FGF9-enriched exosomes as promising therapeutic strategies. Further research and clinical studies are needed to validate these findings and support the development of safe, exosome-based therapies for OA.

## Data Availability

The data that support the findings for this study are available to other researchers from the corresponding author upon reasonable request.
